# Functional Reconstruction of Sarcoma Defects Utilising Innervated Free Flaps

**DOI:** 10.1155/2012/315190

**Published:** 2012-08-28

**Authors:** Damien Grinsell, Claudia Di Bella, Peter F. M. Choong

**Affiliations:** ^1^Department of Plastic Surgery, St. Vincent's Hospital, (Melbourne), Fitzory, VIC 3065, Australia; ^2^Department of Orthopaedics, St. Vincent's Hospital, University of Melbourne, Level 3, Daly Wing, 41 Victoria Parade, Fitzroy, VIC 3065, Australia; ^3^Department of Surgery, The University of Melbourne, Parkville, VIC, Australia

## Abstract

Soft-tissue reconstruction following preoperative radiotherapy and wide resection of soft tissue sarcoma remains a challenge. Pedicled and free tissue transfers are an essential part of limb sparing surgery. We report 22 cases of sarcoma treated with radiotherapy and wide excision followed by one-stage innervated free or pedicled musculocutaneous flap transfers. The resection involved the upper limb in 3 cases, the lower limb in 17, and the abdominal wall in 2. The flaps used for the reconstruction were mainly latissimus dorsi and gracilis. The range of motion was restored fully in 14 patients. The muscle strength of the compartment reconstructed was of grades 4 and 5 in all patients except one. The overall function was excellent in all the cases with functional scores of 71.2% in the upper limb and 84% in the lower limb. The only 2 major complications were flap necrosis, both revised with another flap, one of which was innervated with restoration of function. Innervated flaps are valuable alternatives for reconstruction after sarcoma resection in the extremity and in the abdominal wall. The excellent functional results are encouraging, and we believe that innervated muscle reconstruction should be encouraged in the treatment of sarcoma after radiotherapy and wide resection.

## 1. Introduction

In the mid-1970s the rate of amputation for extremity soft tissue sarcomas was 40–50% [1]. During this period, radiation therapy (RT) was considered a palliative rather than curative modality for the large tumour masses [2]. A decade later, however, Rosenberg and colleagues reported that when compared with amputation, wide excision with external beam RT was associated with equivalent 5-year disease-free and overall survival [3]. Since then, the combination of surgery and RT has been proven to yield superior local control of tumour compared to local excision alone and has been fundamental to the adoption of limb-sparing surgery for high-risk extremity STS [4–7]. Preoperative RT is preferred at our Institute because of smaller RT targets [8, 9]. lower RT dose due to better limb perfusion and oxygenation, [6, 10]. and decreased late toxicity [11] compared with postoperative RT despite a slighter higher rate of wound complications.

Surgical margins are the most important factor associated with local tumour control [12]. In many cases, obtaining good oncologic margins can result in extensive or critical loss of muscle/tendon units. Not only are the reconstructions required after such resections likely to be challenging, wound healing difficulties in the postoperative period [13, 14], especially after radiotherapy [15]. can threaten the viability of any flap. Pedicled and free tissue transfers have been popularised for limb preservation in these difficult cases and have been particularly useful for attaining wound closure, filling surgical dead space, protecting critical structures (i.e., nerves, tendons, and joints), and promoting wound healing [16–18].

The use of innervated flaps has proven to be invaluable in numerous reconstructive procedures (e.g., brachial plexus injury and Volkmann's contracture), and more recently their use has been proposed in soft-tissue sarcomas [19–23]. While sensate reinnervation has been previously studied in orthopaedic reconstructive practice [24], motor innervated muscle transfer is relatively new. Motor re-innervation can provide the dual functions of active contraction and soft tissue coverage, and therefore seems to be ideal for reconstructions after soft tissue sarcomas resection.

This reports on 22 cases of soft-tissue sarcoma treated with radiotherapy followed by one-stage innervated free or pedicled musculocutaneous flap transfers after wide excision. The goal of this study is to evaluate the functional outcome of this procedure and emphasize the usefulness of this procedure in sarcoma surgery even after radiation therapy.

## 2. Patients and Methods

From 2006 to 2010, 112 patients have been treated with neoadjuvant radiotherapy and resection for soft tissue sarcoma followed by reconstruction with free flaps. Of these, a total of 22 had innervated muscle flap reconstructions, and these patients form the focus for this paper. The resection and the flap reconstruction were performed in one stage for all the patients on average mean 6 weeks (range 5–8) after the finish date of preoperative radiotherapy.

The pathology included 14 pleomorphic soft tissue sarcoma, 3 liposarcoma, 1 DFSP, 1 angiosarcoma, 1 fibrosarcoma, 1 chondrosarcoma, and 1 metastatic chondroblastic osteosarcoma with abundant soft tissue extension (Table 1). With the exception of the patient with chondrosarcoma, all the other patients received preoperative radiotherapy (range 50–60 Gy), one patient received preoperative chemotherapy as well.

The resection was wide in all the cases except in one, in which the margins were marginal due to pathologic fracture in metastatic chondroblastic osteosarcoma of the femoral shaft with extension in the soft tissues.

The resection involved the lower limb in 17 patients the upper limb in 3, and the abdominal wall in 2. In the lower limb, four resections involved the leg: the posterior compartment in 3 patients and the anterior compartment in one. In the posterior compartment, gastrocnemius and soleus were resected in 2 patients; flexor digitorum longus, flexor hallucis longus, and soleus in the other patient. The tibialis nerve was included in the resection in all cases. In the anterior compartment, the resection involved tibialis anterior and the deep peroneal nerve (Table 1). The thigh was involved in 13 cases. One patient had a femoral resection and reconstruction with megaprosthesis associated with anterior compartment excision. In all the other cases, the resection involved the soft tissues only; the whole hamstring compartment was excised in 3 patients. In 4 patients the resection involved the posterior compartment of the thigh, and in one included the adductor compartment as well. The adductor compartment of the thigh was excised in 3 patients. In the remaining 2 patients, the entire gluteal compartment was excised (Table 1). In the upper limb, 2 resections involved the anterior compartment of the arm and 1 the rhomboid and trapezius muscles. In the abdomen, a full thickness abdominal wall resection was performed in both cases (Table 1).

The donor flap was selected on the basis of the size of the defect to reconstruct and the size of the overlying skin. Donor flap selection took into account the size of the soft tissue defect including dead space and skin, length of the defect, availability of recipient nerves, and the requirement for a functional reconstruction. An innervated musculocutaneous flap was felt to be indicated when either the whole compartment had been resected or a critical component of joint movement had been severely compromised (i.e., biceps brachii or vastus medialis obliquus with rectus femoris). All flaps included a muscle and skin component apart from the vascularised sural nerve flap. The gracilis myocutaneous flap was used in 7 patients (in 6 innervated, in one the sural nerve was subsequently implanted), the latissimus dorsi in 8 (in 7 cases free, in one pedicled), the TRAM in 3, the tensor fascia lata in 2, the free parascapular with the sural nerve in one and the rectus abdominis in one (Table 1).

The vascular pedicles were anastomosed to recipient vessels available after the tumor resection and all were within the radiotherapy field. The donor nerve of the transferred muscle was sutured using microsurgical techniques and an epineural repair requiring 9/0 or 10/0 nylon. The selection of the recipient nerve was the largest single or multiple motor nerves available after resection was complete. The recipient nerves were stimulated intraoperatively before resection with a hand held nerve stimulator in order to confirm the presence of motor axons.

Postoperatively, the involved limb was immobilized in a splint for 6 weeks; following this time, patients were allowed to start active and passive ROM. Strengthening exercises were commenced after 3–6 months for a minimum period of 12 months.

The mean followup was 17.9 months (range 6–42). The patients were evaluated for:

1. strength in the reconstructed compartment using the MRC scale [25];
2. range of motion (ROM) of the joint(s) controlled by the muscle(s) replaced;
3. overall function using the Lower Extremity Functional Scale (LEFS, 0: unable to perform any activity; 80: excellent function) for the lower limb [26] and the Quick DASH (Disabilities of the Arm, Shoulder and Hand, 0: excellent function; 100: unable to perform any activity) for the upper limb [27];
4. Musculo-Skeletal Tissue Society (MSTS, 0: extremely unsatisfied, 30: extremely satisfied) score [28].

For the two patients who received the abdominal reconstruction, the above evaluation was not performed, and the results were evaluated on the basis of the occurrence of hernia or bulge.

## 3. Results

There were no intraoperative complications. In one patient, there was a superficial infection at the donor site, while in all the others there were no complications at that level.

One patient died of the disease after a subsequent recurrence of the sarcoma which was treated with above knee amputation and was not evaluated.

Of the remaining 21 patients there were a total of 6 (28.5%) postoperative complications referred to the reconstruction, 2 major and 4 minor. The 2 major complications were flap failures in both cases because of necrosis: in one case the original flap (gracilis) was substituted with another innervated flap (lat dorsi); in the other case, the original flap (gracilis) was substituted with a noninnervated VRAM. Of these 2 patients, only the one with the second attempt of innervated flap has been evaluated for functional results. Both cases had preoperative radiotherapy. The four minor complications were lymphoedema in 2 and wound delayed healing in 2. Of these patients, only one required minimal debridement and skin grafting and the other healed with dressings. In all these patients with minor complications the original innervated flap has been preserved and evaluated.

The two patients who received a full-thickness abdominal wall reconstruction regained a complete competency of the abdominal wall and did not develop hernia or bulge. No functional score is available for a detailed evaluation of the results.

A total of 18 patients have been evaluated for functional and emotional results. All of these patients had preoperative radiotherapy, with a dose range of 50 to 60 Gy (mean 57). The response to radiotherapy was good (>90% of necrosis in the final specimen) in 14 patients and poor (<90% of necrosis) in the remaining 4.

**Figure 1:**
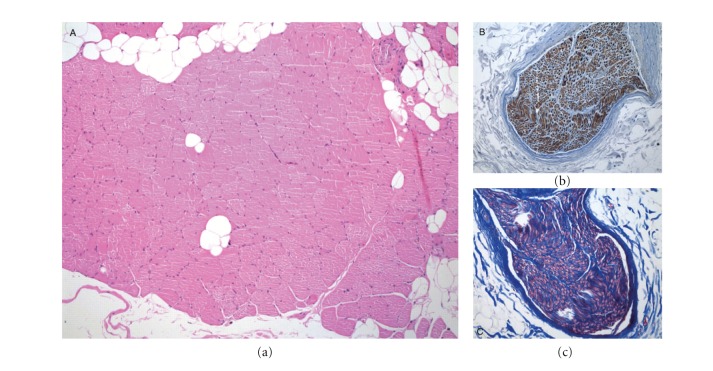
Histology of excised innervated flap (gracilis) after 14 months. (a) Muscle fibres show no features of denervation atrophy, Haematoxylin and Eosin 100x. (b) Nerve section shows normal fasciculi without reactive axons and normal axonal density, APP Immunohistochemistry 200x. (c) Nerve section shows well preserved myelinated fibers axons with normal density, Masson stain 200x.

In one patient (gracilis free flap for adductor compartment resection), a local recurrence was noted and treated with further wide resection and noninnervated flap. The first re-innervated flap was functionally evaluated at 14 months post-operative and is included in the analysis. During the second operation, the first re-innervated flap was resected and histology has been performed to evaluate the status of the muscle and nerve. The muscle showed no features of denervation atrophy and the nerve showed normal myelin sheet and axonal density (Figure 1).

**Figure 2:**
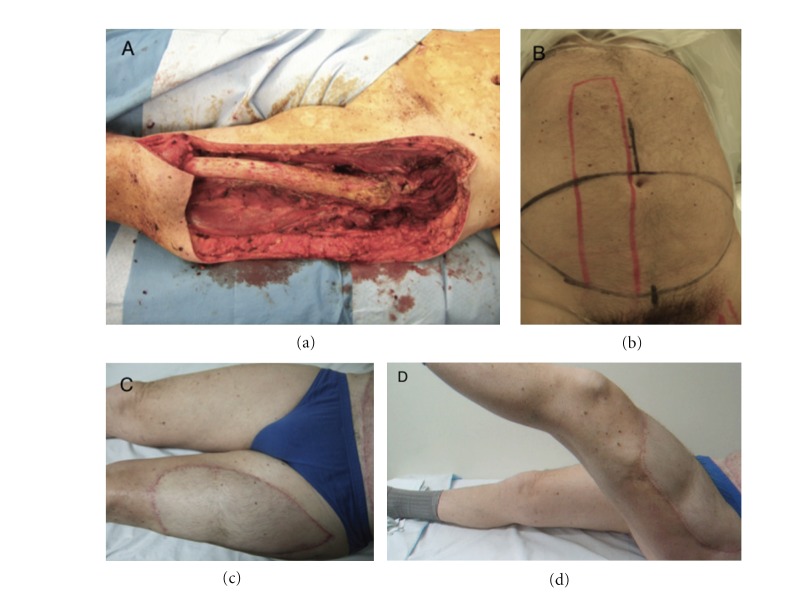
Patient treated for high-grade pleomorphic sarcoma of the anterior compartment of the thigh. (a) Intraoperative image. The resection involved the entire quadriceps compartment and measured 35 × 14 × 25 cm. (b) The reconstruction was performed using innervated TRAM. (c, d) At 24 months postoperatively, the patient demonstrated full extension of the knee, flexion limited to 100 degrees, and muscle strength of 5.

Immediate postoperative strengths in all cases were M0. The strength of the muscle or muscles replaced was M5 in 9 patients at a mean followup of 20.5 months (range 6–42), M4 in 8 patients at a mean followup of 12.8 months (range 6–20) and M3 in 1 at 12 months of followup (Figure 2).

**Figure 3:**
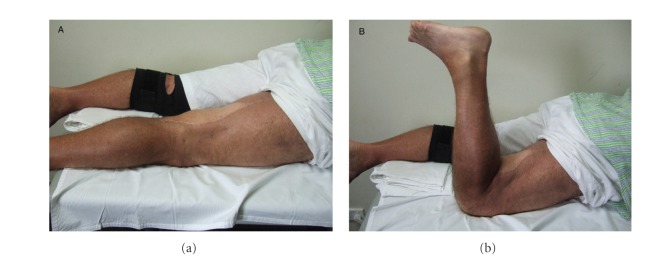
Patient treated for liposarcoma of the posterior thigh with wide resection involving hamstrings, reconstructed with innervated latissimus dorsi flap. Clinical outcome at 14 months post-op. (a) Full extension. (b) Active flexion. Muscle strength of posterior compartment of the thigh: 4.

**Figure 4:**
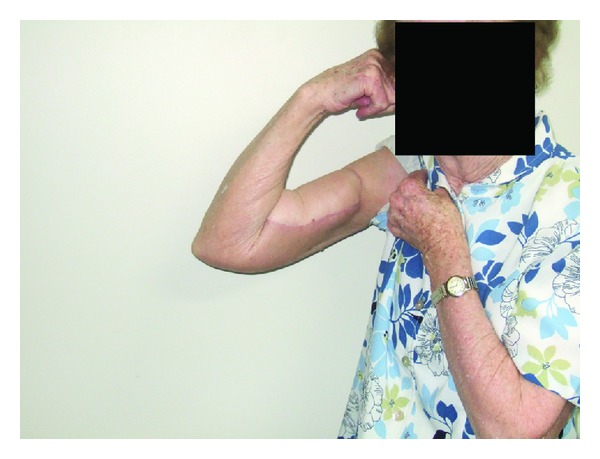
Patient treated for MFH of the anterior compartment of the arm; the resection involved the biceps and the brachialis muscles, and denervation of brachioradialis. The reconstruction has been obtained using free innervated gracilis and transfer of FCU to remaining brachialis. Clinical outcome at 12 months post-op shows full active flexion at the elbow. The muscle strength of the anterior compartment of the arm was 4.

The range of motion (ROM) of the joint or joints controlled totally or partially by the innervated flap was fully restored in 14 patients and partial in 5. In 4 of the cases with partial ROM the joint involved was the knee, with a ROM limited only in flexion up to 70 degrees in one case, 90 in two, and 100 in one. The compartment reconstructed was the posterior of the thigh in 3 cases and the anterior in one (Figure 3). In the other case, the compartment reconstructed was the anterior of the arm, with a ROM limited only in extension and full flexion (ROM 30–140 degrees) (Figure 4).

The overall function was excellent in all the cases. In the three patients in whom the reconstruction involved the upper limb the Quick DASH score were 0, 14 and 31. In the lower limb the mean LEFS was 67.4 (range 31–80).

The mean MSTS score was 27 (range 13–30) (Table 2).

## 4. Discussion

Soft tissue sarcomas are aggressive tumors that require extensive resections to obtain wide margins. Free or pedicled muscle transplantations are often necessary for wound closure, especially when the resection involves a substantial amount of muscle and skin. The main goal of plastic reconstruction has traditionally been soft tissue coverage, because in the majority of the cases the remaining muscles are able to hypertrophy and partially replace the function of the resected muscles [29, 30]. The indication for a functional reconstruction has been limited therefore to the forearm and the posterior leg [30], but in some cases this has been extended to the thigh, the anterior lower leg, the shoulder and the buttock [31]. In this study we showed that these extended indications are appropriate and that, by providing adequate muscle function after tumor resection, the patient's satisfaction and emotional status can be satisfactory.

It is well known that radiation therapy negatively affects microvascular surgery because it causes intimal damage of the vessels. Consequently, lower success rate of the anastomosis has been reported [32, 33], motivating the use of recipient vessels outside the field of irradiation to avoid vascular complication [31]. In agreement with these findings, flap necrosis was seen in only 2 of 21 of our cases. Interestingly, both cases of flap loss were late failures occurring at day 5 and day 15 (after patient was discharged home) suggesting that increased activity may have played a part in the failure. Both were successfully revised with another muscle transfer. Despite the deleterious effects of radiotherapy on flap survival, we showed that it is still possible to perform another reconstructive limb salvage procedure with satisfactory results. Moreover, in one of the two patients we were able to implant another re-innervated flap and therefore maintaining the chance of a functional reconstruction.

The complication rate after free or pedicled musculocutaneous flap reconstruction may be higher compared to primary closure and this is mainly due to the fact that patients receiving flaps have a significantly larger and higher-grade tumors than patients treated with primary closure, and were more likely to have received preoperative irradiation, bone resection, and motor nerve resection [34]. In our experience with large sarcoma defects even if primary closure is possible we have witnessed increased rates of haematoma, seroma, and wound breakdowns due to the deadspace and radiotherapy and now favour importation of well vascularised tissue in the form of free flaps. Each of these tumour and treatment factors has been associated with worse function and/or health status outcomes [35–37]. At our institution, the use of innervated flaps did not increase the amount or the severity of post-operative complications compared to noninnervated flaps, while providing a much better functional outcome. We agree with other authors that the time of the surgery and the amount of blood loss are not influenced by the use of an innervated flap compared with non-functional flaps [31]; Whilst the surgeon experience required to perform an innervated flap is the same required to perform a normal free flap and requires no further training, the complexity of including multiple vessel and nerve repairs and tensioning of muscle and tendon units makes it a more complex task. We believe, however, that the excellent functional outcome for these patients justifies the potentially higher flap loss rate.

The main limit of this study is the small number of patients and the short-term followup. Further larger studies are necessary to compare this reconstruction with non-innervated muscle flaps.

## 5. Conclusion

Re-innervated flaps are viable options for reconstruction after soft tissue tumor resection of the extremity. The functional results are encouraging and we believe that the indication for re-innervated muscle reconstruction can include both upper and lower limb. Longer-term studies and comparative studies are necessary to better understand the most appropriate indications for this type of reconstruction.

## Figures and Tables

**Table 1:** Patients distributions regarding histology, compartment involved in the resection, muscle function replaced and flap used.

Patient	Histology	Compartment resected	Muscle function replaced	Flap used
1	Dermatofibrosarcoma protuberans	Leg, posterior	Soleus + flexor hallucis longus + flexor digitorum longus	Gracilis + suraln. (second stage)
2	Pleiomorphic sarcoma (recurrent)	Hip, extensor	Gluteus max	TRAM
3	Pleiomorphic sarcoma	Thigh, adductor	Adductors	Gracilis
4	Chondroblastic OS (metastatic)	Thigh, extensor	Quadriceps	Latissimus dorsi
5	Pleiomorphic sarcoma	Arm, anterior	Biceps + brachialis	Gracilis (failed, substituted with pedicled lat dorsi)
6	Pleiomorphic sarcoma	Thigh, posterior	Hamstrings	Latissimus dorsi
7	Neurofibrosarcoma	Leg, posterior	Soleus + gastrocnemius	Parascapular + suraln.
8	Angiosarcoma	Thigh, extensor	Entire VMO + rectus femoris	Gracilis
9	Liposarcoma	Thigh, posterior and adductor	Hamstrings + adductor magnus	Latissimus dorsi
10	Liposarcoma	Scapular stabilizers	Rhomboid + trapezius	Latissimus dorsi
11	Pleiomorphic sarcoma	Arm, anterior	Biceps + brachialis	Gracilis
12	Pleiomorphic sarcoma	Thigh, anterolateral	Vastus lateralis + rectus femoris	TRAM
13	Liposarcoma	Leg, anterior	Tibialis, anterior	Gracilis
14	Pleiomorphic sarcoma	Thigh, adductor	Adductors	Gracilis
15	Pleiomorphic sarcoma	Thigh, posterior	Hamstrings	Latissimus dorsi
16	Pleiomorphic sarcoma	Hip extensors	All Gluteal muscles	Latissimus dorsi
17	Pleiomorphic sarcoma (recurrent)	Thigh, posterior	Hamstrings	Latissimus dorsi
18	Pleiomorphic sarcoma	Thigh, adductor	Adductors	Rectus Abdominis
19	Fibrosarcoma	Leg, posterior	Soleus + gastrocnemius	Latissimus dorsi
20	Pleiomorphic sarcoma	Thigh, anterior	Quadriceps	TRAM
21	Chondrosarcoma	Flank	Abdominal wall + iliac crest	Tensor fascia lata
22	Pleiomorphic sarcoma	Flank	Abdominal wall	Tensor fascia lata

**Table 2:** Outcome of the innervated flaps.

Patient	Followup (months)	Complications	Muscle strength	DASH/LEFS	MSTS
1	42	Wound breakdown	M5	69	27
2	19	—	M5	80	30
3	Died of disease	—	—	—	—
4	(1)	Flap failure (necrosis at 1 month po), substituted with no innervated flap	—	—	—
5	15	Flap failure (necrosis at 5 days po), substituted with reinnervated lat. dorsi	M4	*31*(DASH)	25
6	17	Lymphoedema	M4	47	23
7	20	Lymphoedema + superficial infection	M4	31	30
8	20	—	M5	80	30
9	14	—	M4	52	27
10	14	—	M5	0 (DASH)	30
11	15	—	M4	14 (DASH)	13
12	24	Superficial infection at donor site	M5	57	30
13	24	—	M5	78	30
14	18	—	M5	73	26
15	6	—	M4	75	27
16	6	—	M5	80	30
17	8	—	M4	80	30
18	8	—	M4	65	26
19	12	—	M3	65	25
20	18	—	M5	80	30
21	20	—	—	—	—
22	23	—	—	—	—
